# Supplementary material for: Long-term exposure to ambient PM2.5, particulate constituents and hospital admissions from non-respiratory infection
Source: Nat Commun. 2024 Feb 19;15:1518. doi: 10.1038/s41467-024-45776-0 (PMC10876532; doi:10.1038/s41467-024-45776-0)
Supplement: Supplementary file 1 — Supplementary Information [file 41467_2024_45776_MOESM1_ESM.pdf]

Supplementary information

Supplementary methods

Weighted quantile sum regression

The generalized linear weighted quantile sum regression proceeds in steps. The original data is divided into two groups. Then each of the exposures is converted into a categorical variable representing the quantiles (in our case deciles) of that exposure. In the first group a regression (in our case quasi-Poisson) is fit relating the outcome to the quantiles of all of the exposures. Since all exposures are on the same scale (deciles) the coefficients of each exposure can be interpreted as the relative weight of a 1 decile increase in that exposure in predicting the outcome. The weights are scaled to sum to one. Then, using those weights, in the held-out data set a new variable is computed that is the weighted sum of each of the exposures (as deciles). This single variable is then used as the exposure in a regression (quasi-Poisson, in our case) predicting the outcome. Both regression control for all covariates. In weighted quantile sum regression, the threshold for the most influential components was defined as weight > (1/number of species) (1)

Rescaling the source factors

For each ZIP code in each year, we first calculated a total PM2.5 mixture level by adding up the concentration of the 15 constituents. Within each of the 9 strata defined by time period and mixture pattern, we obtained the concentration of source factors (either 4 or 5 factors within each stratum) for the ZIP code-year. For a typical stratum with 4 factors, we run the regression model below

$$\textit{total PM2.5 mixture} = \beta_0 + \beta_1 \times factor1 + \beta_2 \times factor2 + \beta_3 \times factor3 + \beta_4 \times factor4$$

The concentration of each source factor was multiple by its corresponding β and converted to concentration with unit as μg/m^3. After the conversion, we compiled all the source factor data across strata and calculated the standard deviation for each of the source-specific PM2.5. Concentration of source-specific PM2.5 was rescaled by dividing by the source-specific standard deviation. The standard deviation for PM2.5 sourced from oil combustion, soil, coal burning, traffic, biomass burning, and regionally transported nitrates were 0.72, 0.68, 1.91, 1.37, 1.03 and 1.37 respectively.

Random effect meta-analysis

We used random effect meta-analysis to combine the stratum-specific effect estimates of the source-specific PM2.5(2). In random effect analysis, we assume that the mean effects are different across strata defined by time and region. Within each of the strata, the effect estimated is assumed to be

$$\hat{\theta}_i = \mu + \gamma_i + \epsilon_i$$

$$\hat{\theta}_i \sim N(0, \tau^2 + \hat{\sigma}_i^2)$$

Where μ is the population effect; γ_i is the random effects for each stratum and follow the distribution below;

$$\gamma_i \sim N(0, \tau^2)$$

While ϵ_i is the statistical error in the estimates of stratum-specific effect and follows

$$\epsilon_i \sim N\left(0, \hat{\sigma}_i^2\right)$$

The population effect of each source-specific PM2.5 $\hat{\mu}$ is estimated by

$$\hat{\mu} = \frac{\sum_{i=1}^{k} \frac{1}{\hat{\tau}^2 + \hat{\sigma}_i^2} \hat{\theta}_i}{\sum_{i=1}^{k} \frac{1}{\hat{\tau}^2 + \hat{\sigma}_i^2}}$$

Where $\hat{\tau}^2$ and $\hat{\sigma}_i^2$ are the maximum likelihood estimator of the random effects and statistical error.

Identification of source factors

The elemental components of PM2.5 mass usually originated from different sources. Specific elements or groups of elements that are highly associated with a source of air pollution could be serve as tracers and help us identify the sources of air pollution(3). From non-negative matrix factorization, we obtain the loadings of each element (constituent) within each factor and the results are shown in figure S6-S14. We identified the source of each factor based on the loadings of tracer elements. For example, if a factor A has high loadings of Ni and V, then factor A is identified as oil combustion. The tracer elements for each source of air pollution are listed below:

Sources	Tracers
Oil combustion	Ni, V
Soil, dirt	Ca, Si
Coal burning	SO4
Traffic	EC, Zn, Pb, Cu, Fe
Biomass	K
Regionally transported nitrate	NH4

Supplementary table 1. Distribution of coefficient of variation of air pollution data for each ZIP code across the study period.

	Coefficient of variation
	Median (25th%, 75th%)
PM2.5	5.5% (3.6%, 8.6%)
Br	3.5% (2.5%, 4.9%)
Ca	6.9% (5.3%, 9.2%)
Cu	14.1% (8.9%, 21.6%)
EC	9.6% (6.9%, 13.4%)
Fe	7.0% (5.0%, 10.0%)
K	5.8% (3.9%, 8.7%)
NH4	6.2% (4.8%, 8.4%)
Ni	20.9% (13.6%, 33.6%)
NO3	8.1% (5.6%, 12.0%)
OC	6.3% (4.3%, 9.1%)
Pb	10.3% (7.7%, 13.9%)
Si	6.9% (4.9%, 8.2%)
SO4	3.8% (2.8%, 5.0%)
V	11.9% (8.0%, 18.0%)
Zn	8.4% (6.1%, 11.9%)

Supplementary Figure S1. Weights of PM2.5 constituents in the mixture index for the association between the mixture and central nervous system infections.

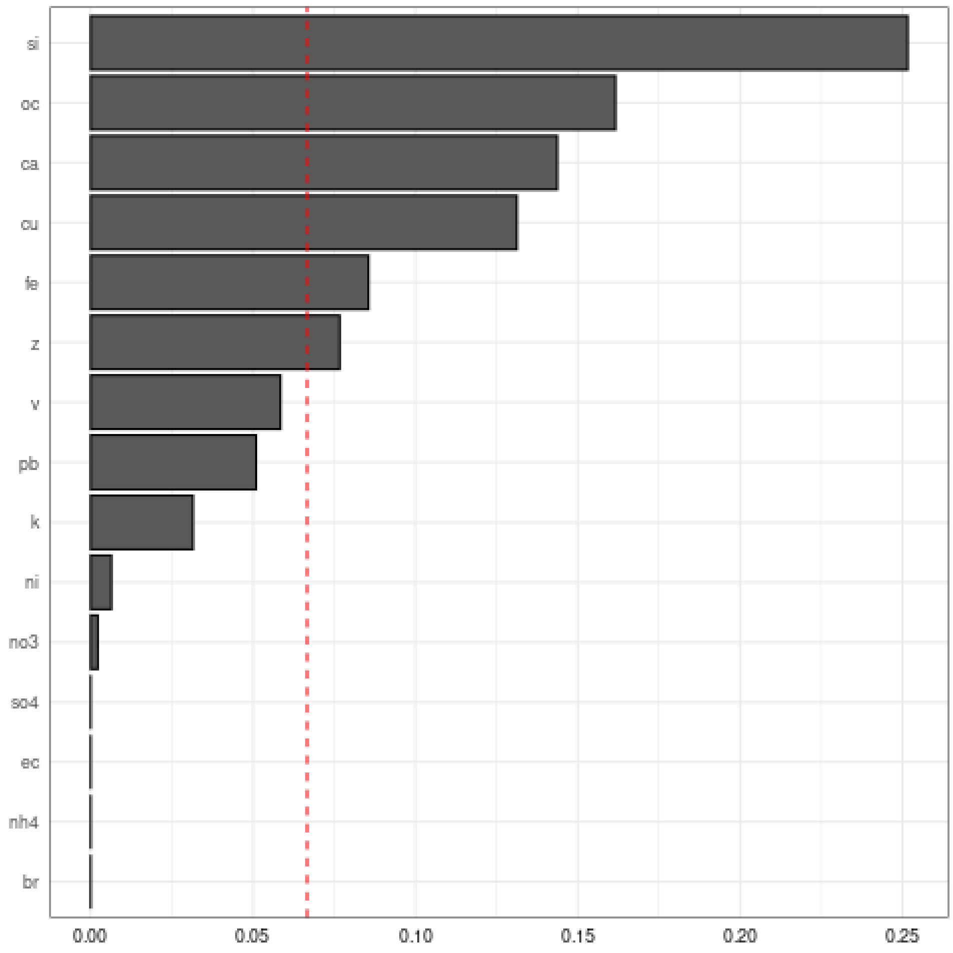

The dash line indicated the threshold for the suggested threshold for the most influential constituents. Source data are provided as a Source Data file.

Supplementary Figure S2. Weights of PM2.5 constituents in mixture index for the association between mixture and intestinal infections.

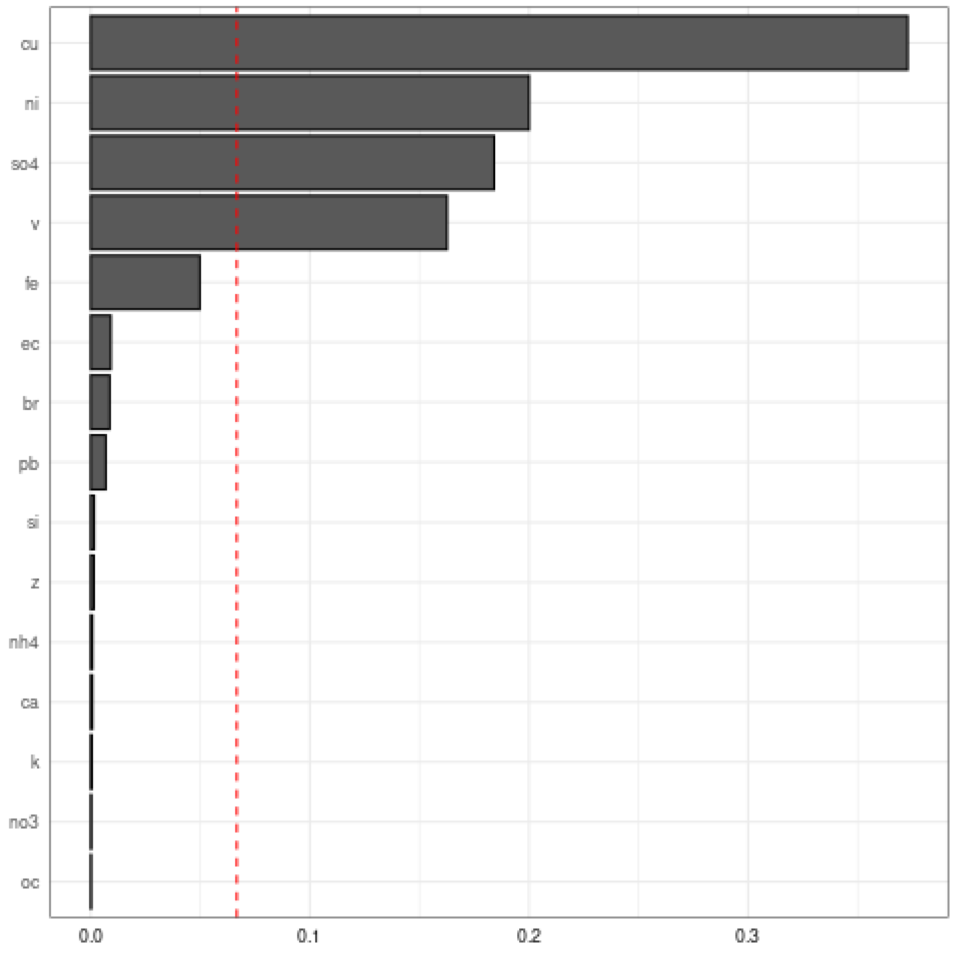

The dash line indicated the suggested threshold for the most influential constituents. Source data are provided as a Source Data file.

Supplementary Figure S3. Weights of PM2.5 constituents in the mixture index for the association between the mixture and urinary tract infections.

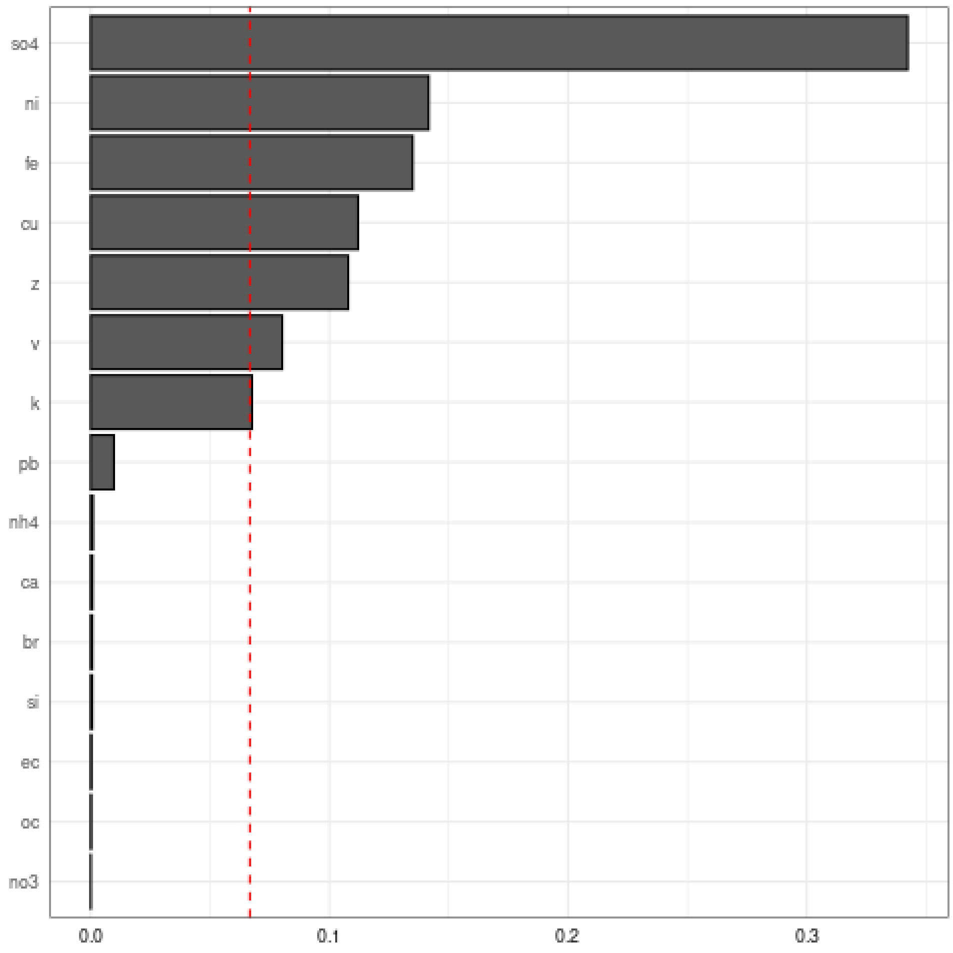

The dash line indicated the suggested threshold for the most influential constituents. Source data are provided as a Source Data file.

Supplementary Figure S4. Weights of PM2.5 constituents in the mixture index for the association between the mixture and septicemia.

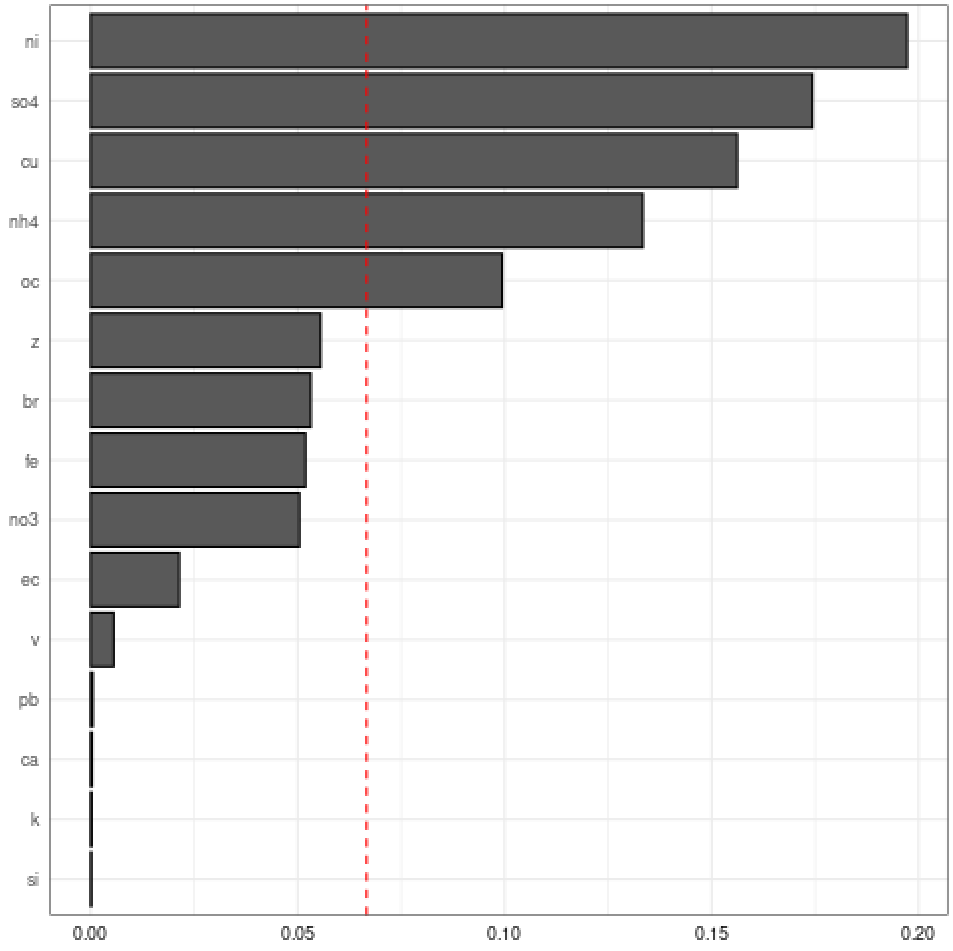

The dash line indicated the suggested threshold for the most influential constituents. Source data are provided as a Source Data file.

Supplementary Figure S5. Clusters of ZIP codes within which separated non-negative matrix factorizations were conducted for the PM2.5 constituent data between 2000-2005.

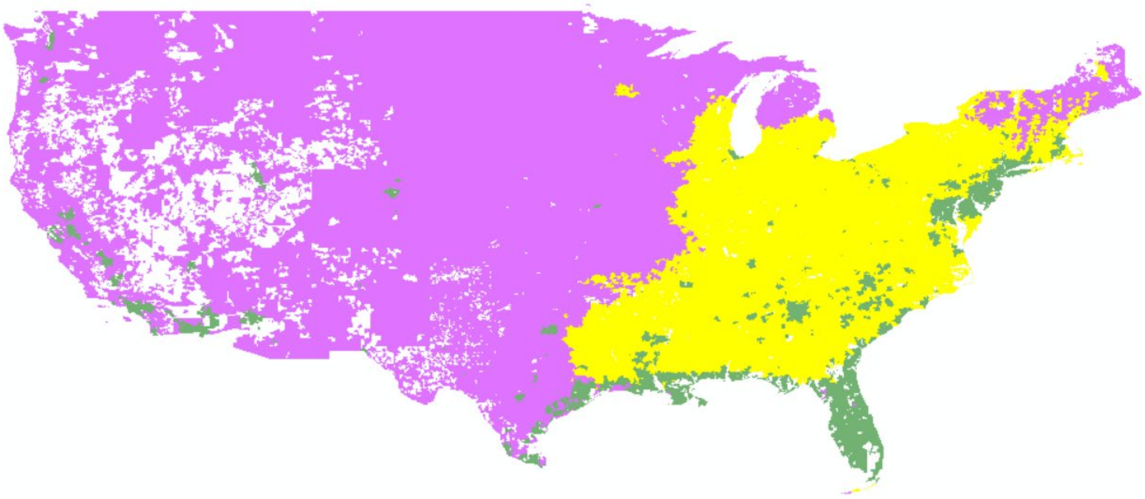

Purple area indicates the regions included in Cluster 1 between 2000-2005; yellow area indicates the regions included in Cluster 2 between 2000-2005; green area indicates the regions included in Cluster 3 between 2000-2005. Source data are provided as a Source Data file. The figure was created from ArcMap 10.7.

Supplementary Figure S6. Clusters of ZIP codes within which separated non-negative matrix factorizations were conducted for the PM2.5 constituent data between 2006-2010.

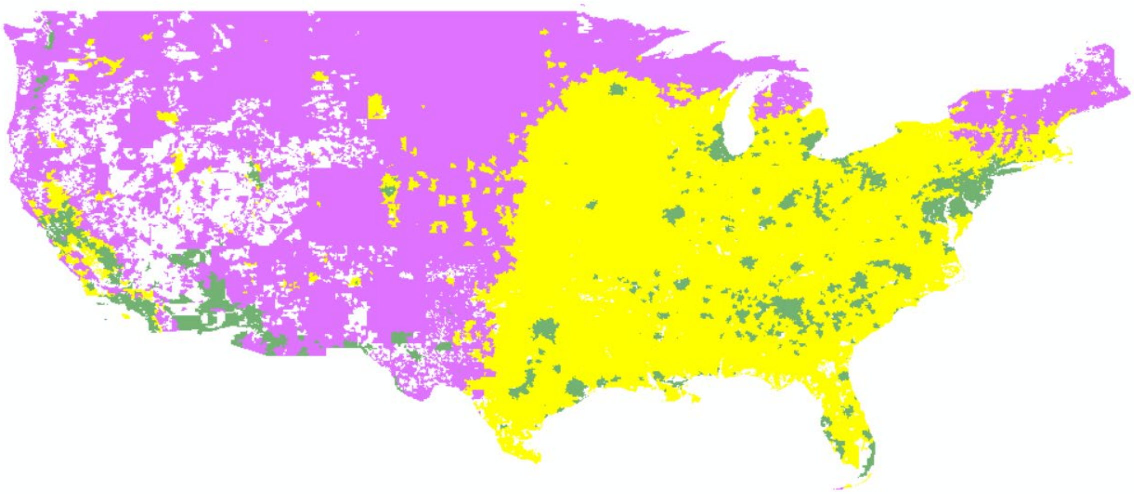

Purple area indicates the regions included in Cluster 1 between 2006-2010; yellow area indicates the regions included in Cluster 2 between 2006-2010; green area indicates the regions included in Cluster 3 between 2006-2010. Source data are provided as a Source Data file. The figure was created from ArcMap 10.7.

Supplementary Figure S7. Clusters of ZIP codes within which separated non-negative matrix factorizations were conducted for the PM2.5 constituent data between 2011-2016.

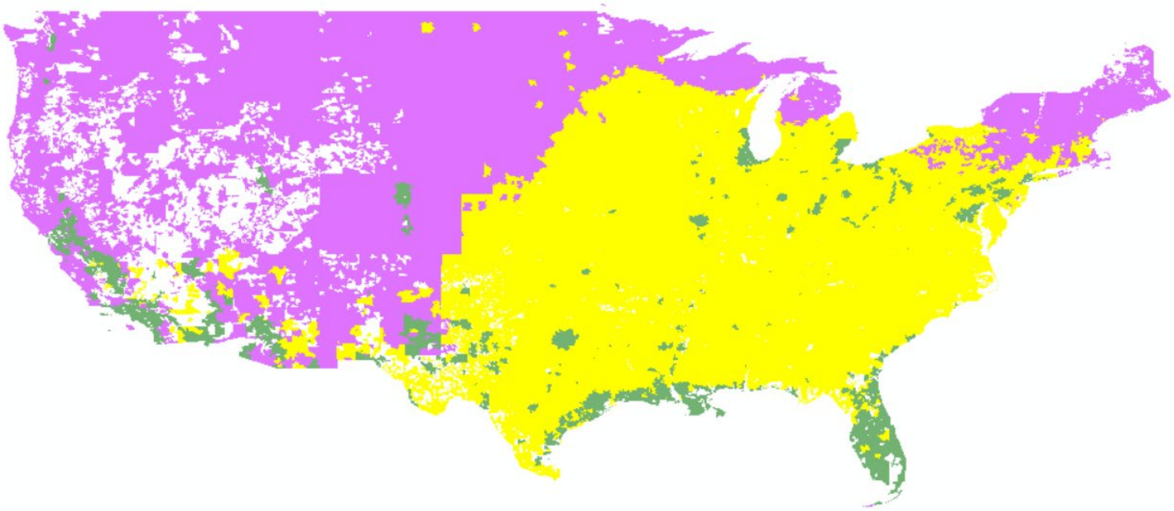

Purple area indicates the regions included in Cluster 1 between 2011-2016; yellow area indicates the regions included in Cluster 2 between 2011-2016; green area indicates the regions included in Cluster 3 between 2011-2016. Source data are provided as a Source Data file. The figure was created from ArcMap 10.7

Supplementary Figure S8. The heatmap of loadings of each PM2.5 constituents on each factor obtained from non-negative matrix factorization in cluster 1 between 2000-2005. Basis 1 was identified as oil combustion; basis 2 was identified as dirt; basis 3 was identified as coal burning; basis 4 was identified as traffic.

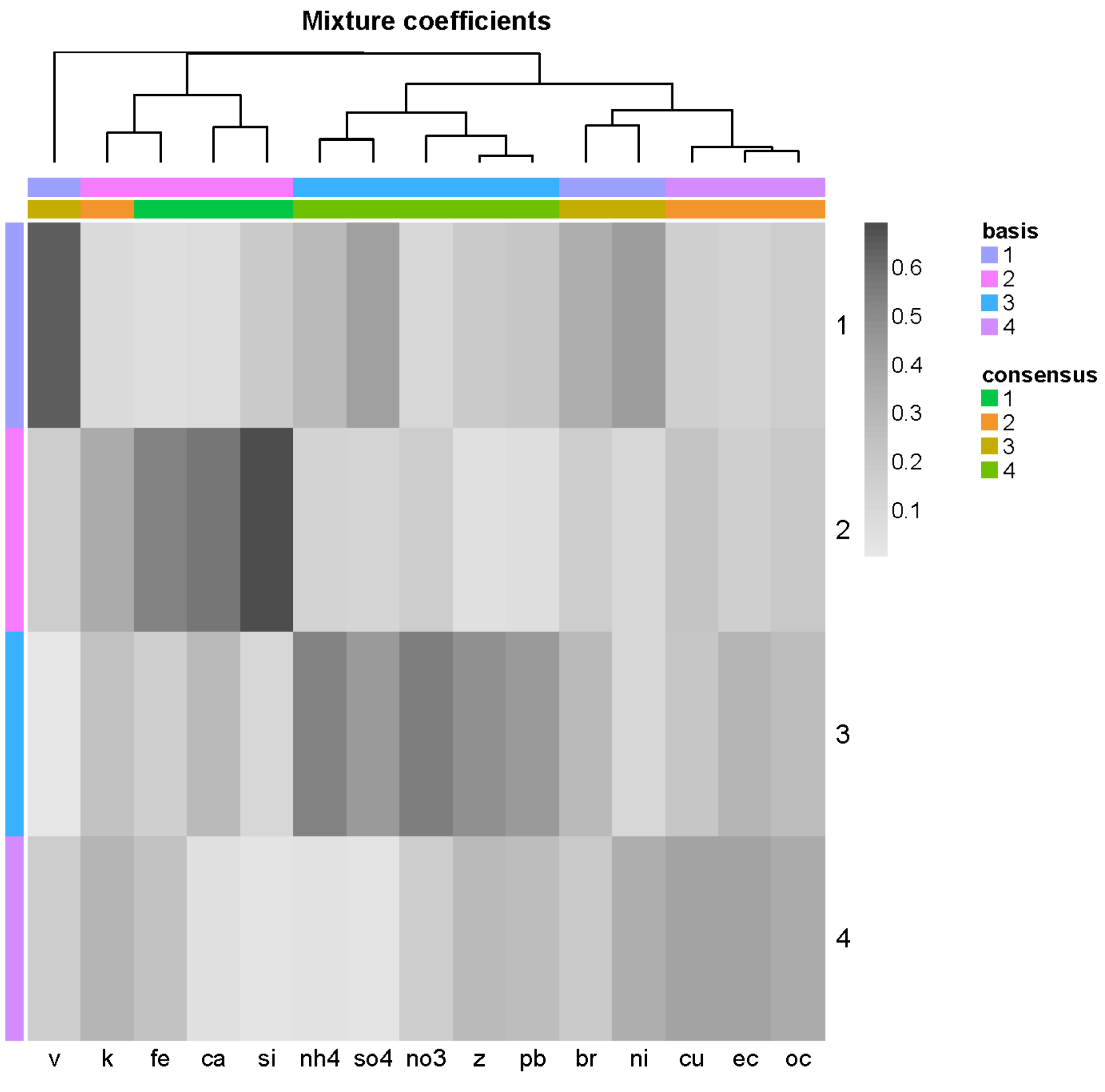

The numbers (1-4) on the Y axis indicates each of the source factors while the grey scale indicates the loading of each component in each source factor. Basis indicates the number of factors identified from

non-negative matrix factorization (NMF). Consensus indicates the consensus across multiple runs of NMF. Source data are provided as a Source Data file.

Supplementary Figure S9. The heatmap of loadings of each PM2.5 constituents on each factor obtained from non-negative matrix factorization in cluster 2 between 2000-2005. Basis 1 was identified as coal burning; basis 2 was identified as oil combustion; basis 3 was identified as dirt; basis 4 was identified as traffic.

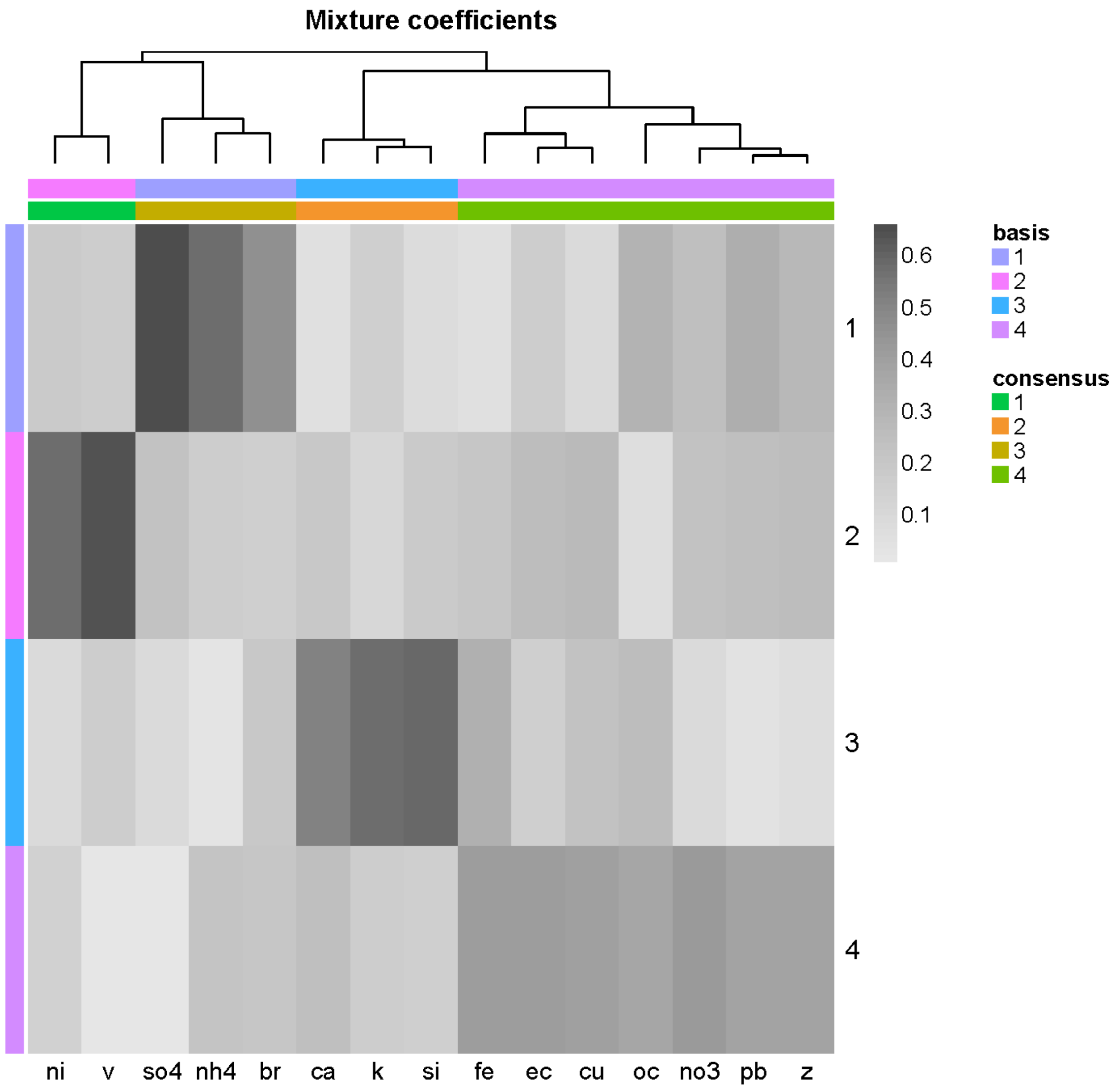

The numbers (1-4) on the Y axis indicates each of the source factors while the grey scale indicates the loading of each component in each source factor. Basis indicates the number of factors identified from

non-negative matrix factorization (NMF). Consensus indicates the consensus across multiple runs of NMF. Source data are provided as a Source Data file.

Supplementary Figure S10. The heatmap of loadings of each PM2.5 constituents on each factor obtained from non-negative matrix factorization in cluster 3 between 2000-2005. Basis 1 was identified as oil combustion; basis 2 was identified as traffic; basis 3 was identified as biomass burning; basis 4 was identified as coal burning.

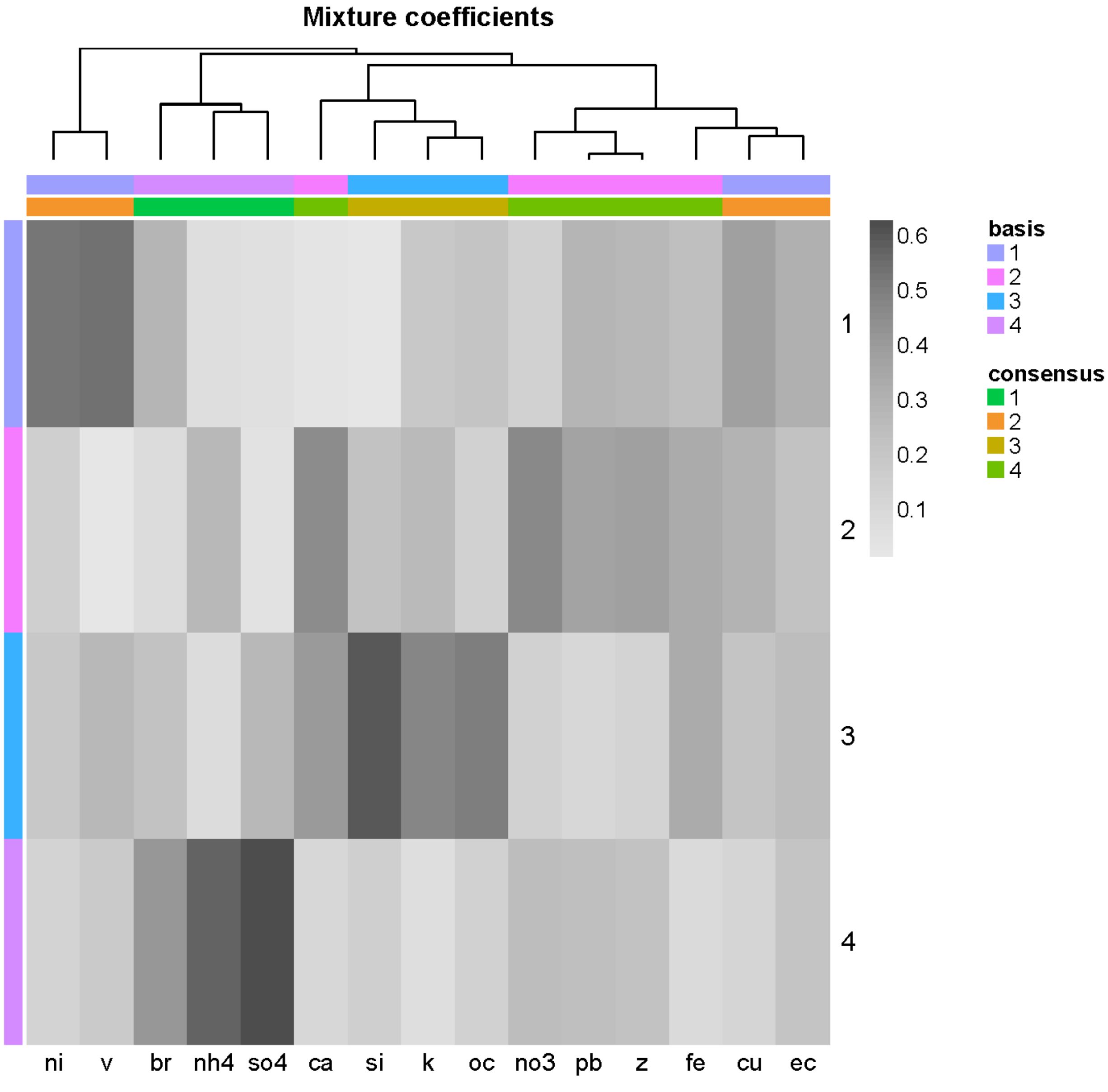

The numbers (1-4) on the Y axis indicates each of the source factors while the grey scale indicates the loading of each component in each source factor. Basis indicates the number of factors identified from

non-negative matrix factorization (NMF). Consensus indicates the consensus across multiple runs of NMF. Source data are provided as a Source Data file.

Supplementary Figure S11. The heatmap of loadings of each PM2.5 constituents on each factor obtained from non-negative matrix factorization in cluster 1 between 2006-2010. Basis 1 was identified as mix of biomass burning and traffic; basis 2 was identified as dirt; basis 3 was identified as coal burning; basis 4 was identified as oil combustion.

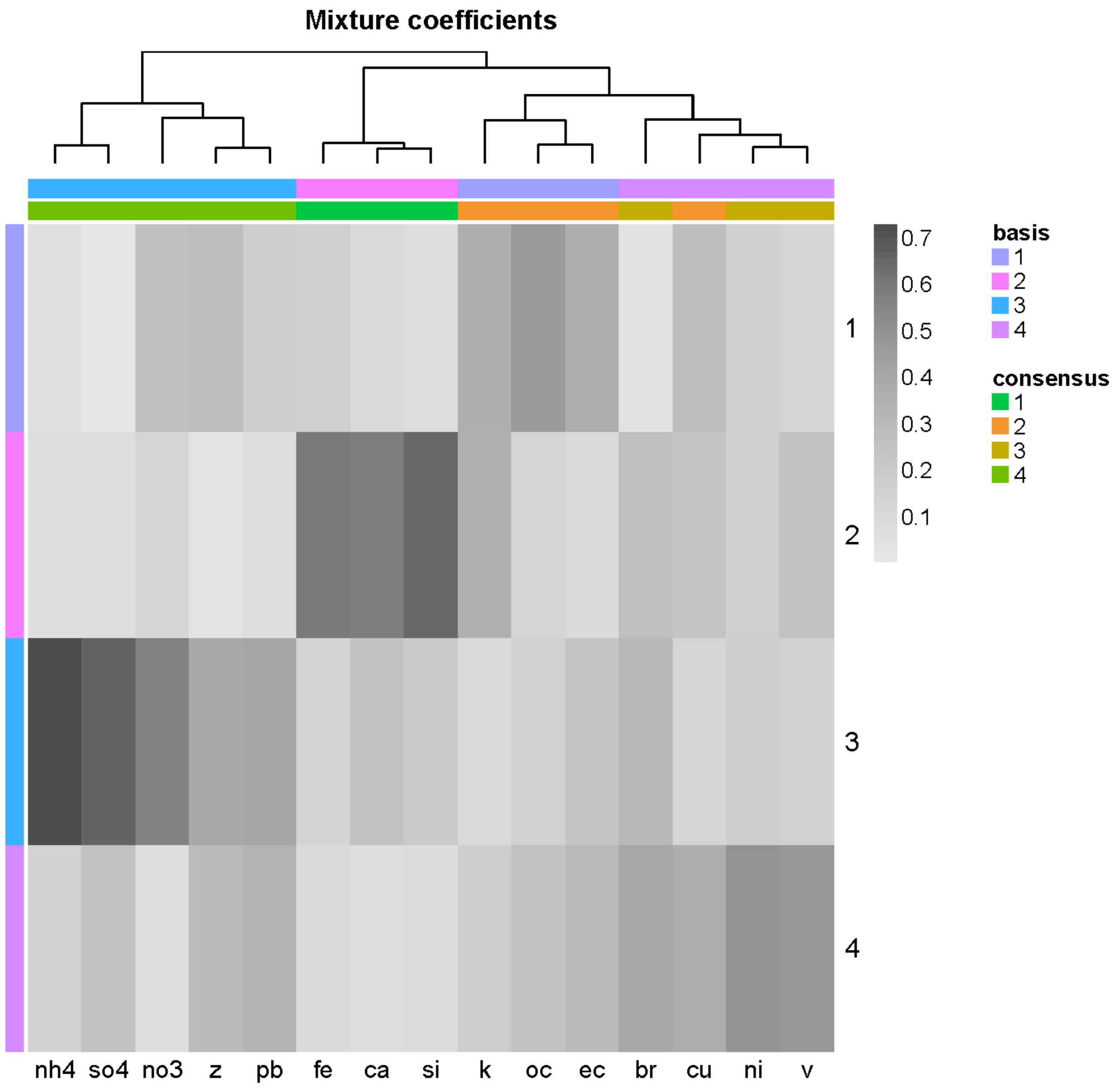

The numbers (1-4) on the Y axis indicates each of the source factors while the grey scale indicates the loading of each component in each source factor. Basis indicates the number of factors identified from

non-negative matrix factorization (NMF). Consensus indicates the consensus across multiple runs of NMF. Source data are provided as a Source Data file.

Supplementary Figure S12. The heatmap of loadings of each PM2.5 constituents on each factor obtained from non-negative matrix factorization in cluster 2 between 2006-2010. Basis 1 was identified as traffic; basis 2 was identified as oil combustion; basis 3 was identified as dirt; basis 4 was identified as coal burning.

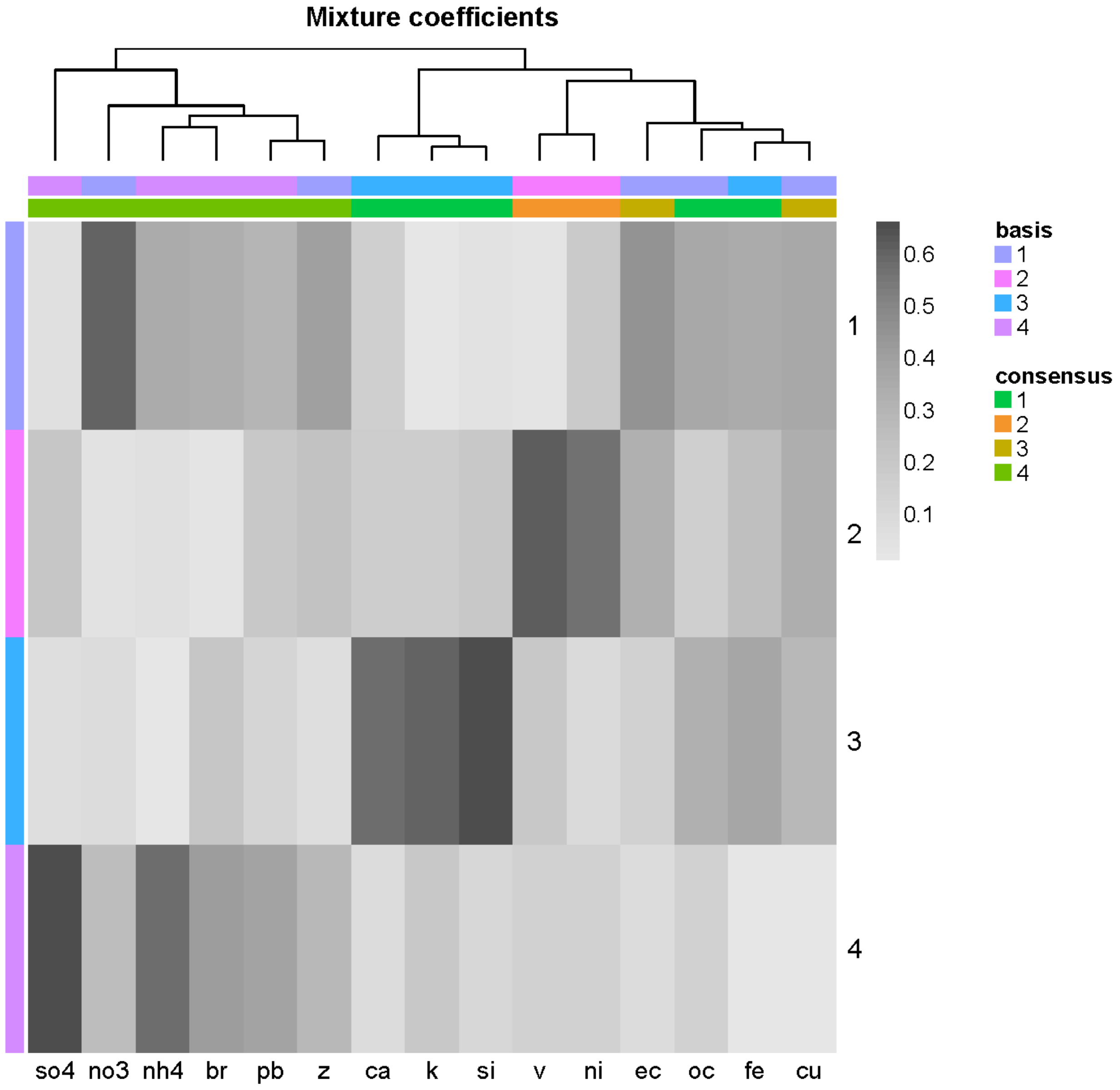

The numbers (1-4) on the Y axis indicates each of the source factors while the grey scale indicates the loading of each component in each source factor. Basis indicates the number of factors identified from

non-negative matrix factorization (NMF). Consensus indicates the consensus across multiple runs of NMF. Source data are provided as a Source Data file.

Supplementary Figure S13. The heatmap of loadings of each PM2.5 constituents on each factor obtained from non-negative matrix factorization in cluster 3 between 2006-2010. Basis 1 was identified as oil combustion; basis 2 was identified as coal burning; basis 3 was identified as regionally transported nitrate; basis 4 was identified as biomass burning; basis 5 was identified as traffic

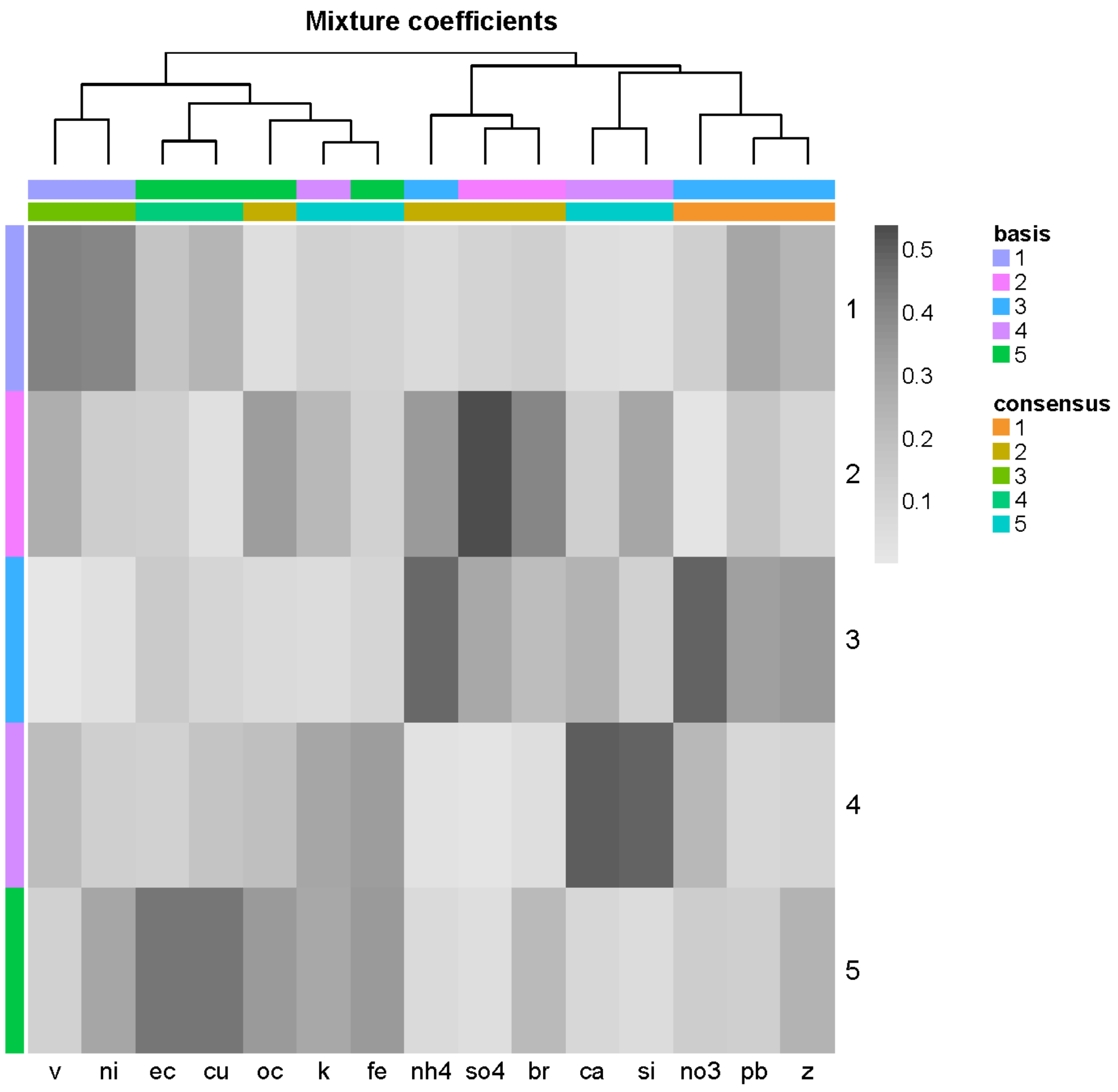

The numbers (1-5) on the Y axis indicates each of the source factors while the grey scale indicates the loading of each component in each source factor. Basis indicates the number of factors identified from

non-negative matrix factorization (NMF). Consensus indicates the consensus across multiple runs of NMF. Source data are provided as a Source Data file.

Supplementary Figure S14. The heatmap of loadings of each PM2.5 constituents on each factor obtained from non-negative matrix factorization in cluster 1 between 2011-2016. Basis 1 was identified as oil combustion; basis 2 was identified as coal burning; basis 3 was identified as traffic; basis 4 was identified as dirt; basis 5 was identified as regionally transported nitrate.

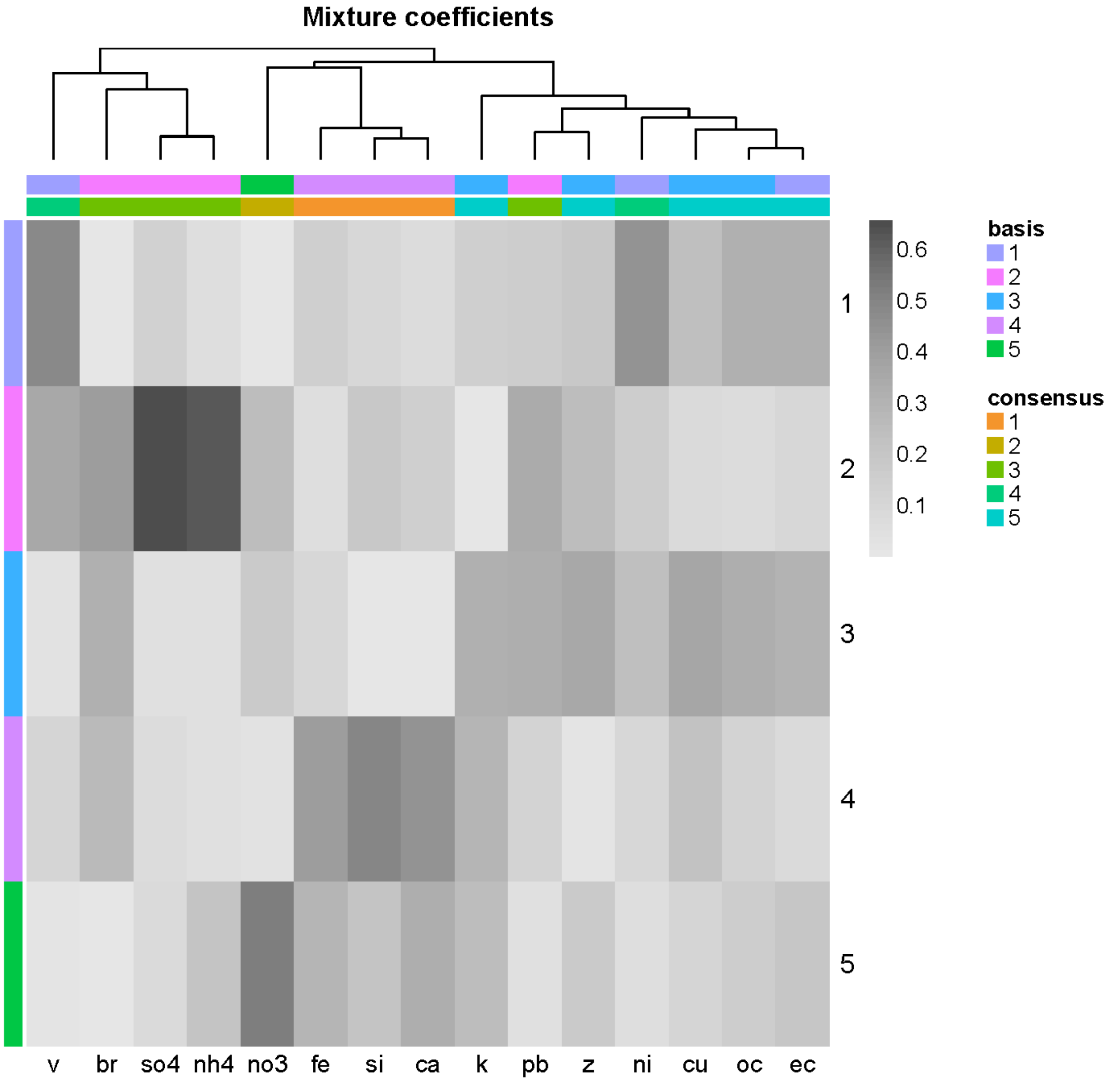

The numbers (1-5) on the Y axis indicates each of the source factors while the grey scale indicates the loading of each component in each source factor. Basis indicates the number of factors identified from

non-negative matrix factorization (NMF). Consensus indicates the consensus across multiple runs of NMF. Source data are provided as a Source Data file.

Supplementary Figure S15. The heatmap of loadings of each PM2.5 constituents on each factor obtained from non-negative matrix factorization in cluster 2 between 2011-2016. Basis 1 was identified as dirt; basis 2 was identified as oil combustion; basis 3 was identified as non-tailpipe traffic; basis 4 was identified as coal burning; basis 5 was identified as tailpipe traffic.

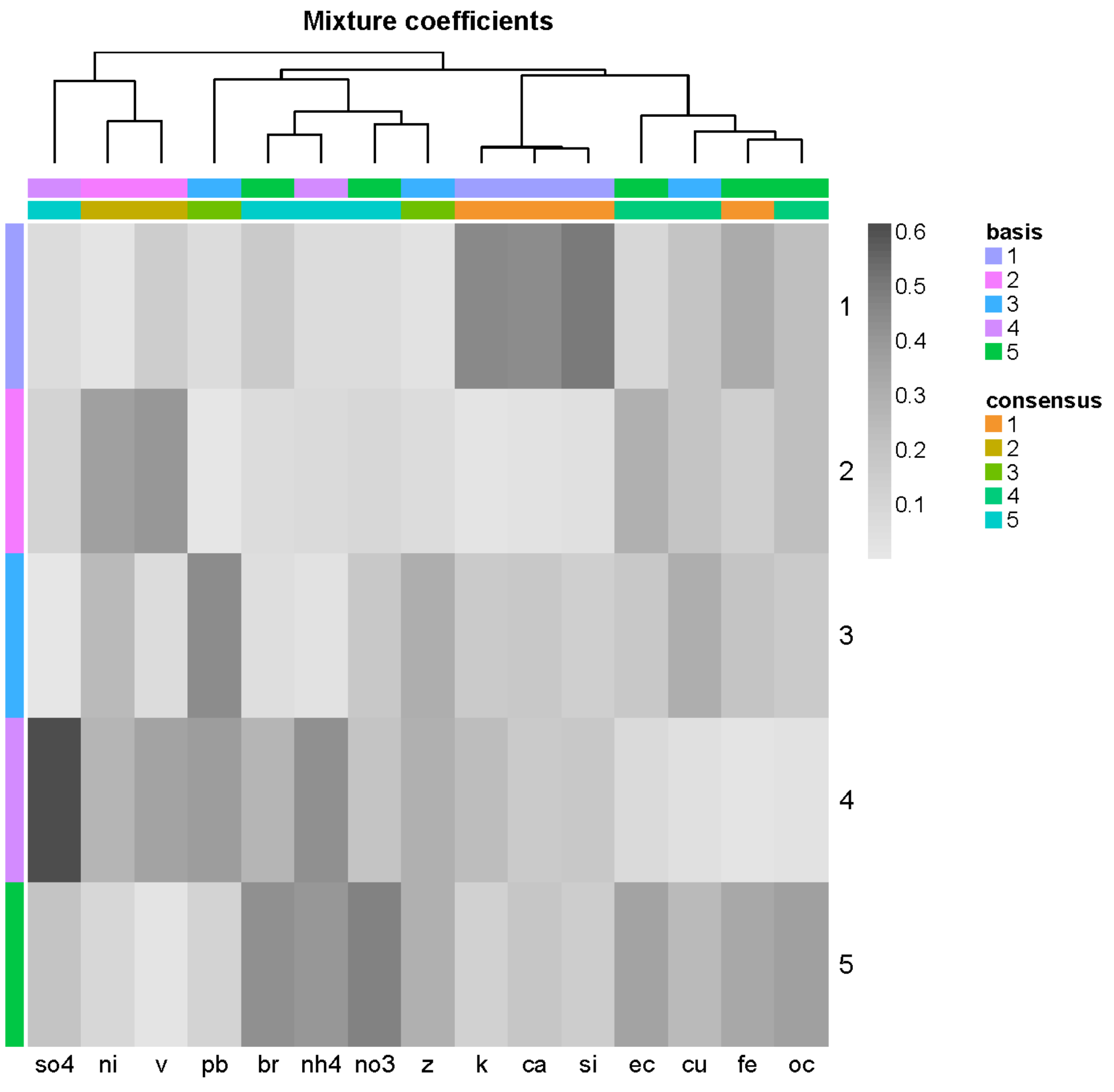

The numbers (1-5) on the Y axis indicates each of the source factors while the grey scale indicates the loading of each component in each source factor. Basis indicates the number of factors identified from

non-negative matrix factorization (NMF). Consensus indicates the consensus across multiple runs of NMF. Source data are provided as a Source Data file.

Supplementary Figure S16. The heatmap of loadings of each PM2.5 constituents on each factor obtained from non-negative matrix factorization in cluster 3 between 2011-2016. Basis 1 was identified as biomass burning; basis 2 was identified as dirt; basis 3 was identified as regionally transported nitrate; basis 4 was identified as traffic; basis 5 was identified as coal burning.

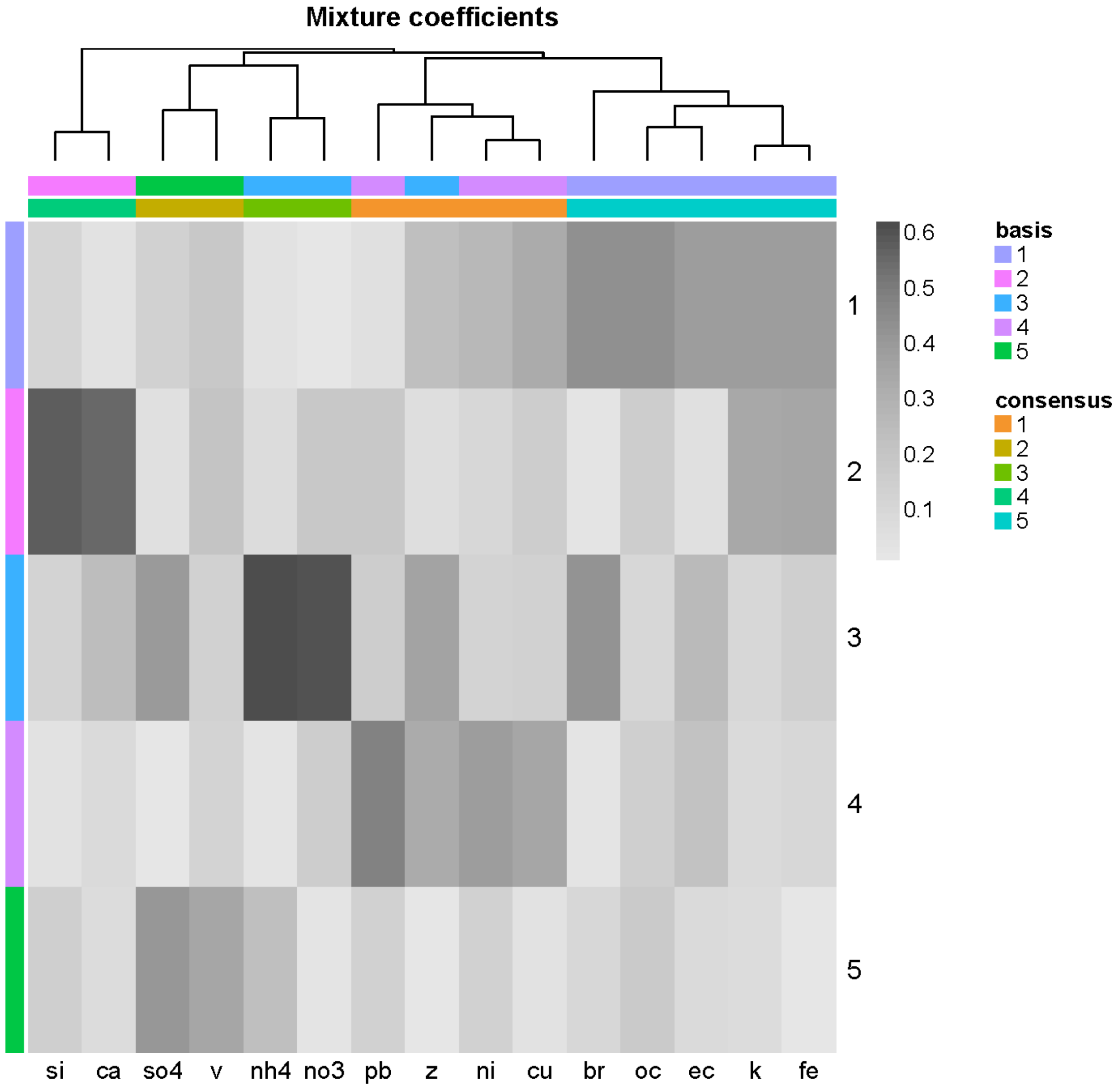

The numbers (1-5) on the Y axis indicates each of the source factors while the grey scale indicates the loading of each component in each source factor. Basis indicates the number of factors identified from

non-negative matrix factorization (NMF). Consensus indicates the consensus across multiple runs of NMF. Source data are provided as a Source Data file.

References

1. Carrico C, Gennings C, Wheeler DC, Factor-Litvak P. Characterization of Weighted Quantile Sum Regression for Highly Correlated Data in a Risk Analysis Setting. J Agric Biol Environ Stat. 2015;20(1):100-20.
2. Higgins JP, Thompson SG, Spiegelhalter DJ. A re-evaluation of random-effects meta-analysis. J R Stat Soc Ser A Stat Soc. 2009;172(1):137-59.
3. Thurston GD, Ito K, Lall R. A Source Apportionment of U.S. Fine Particulate Matter Air Pollution. Atmos Environ (1994). 2011;45(24):3924-36.
