# Supplementary material for: Long-term exposure to ambient PM2.5, particulate constituents and hospital admissions from non-respiratory infection
Source: Nat Commun. 2024 Feb 19;15:1518. doi: 10.1038/s41467-024-45776-0 (PMC10876532; doi:10.1038/s41467-024-45776-0)
Supplement: Supplementary file 3 — Description of Additional Supplementary Files [file 41467_2024_45776_MOESM3_ESM.pdf]

Description of Additional Supplementary Files

Title: Supplementary Code

Description: The supplementary code file includes the analytic code for this analysis and a demo dataset. The real data for this study is restricted from share. Therefore, the demo data is just a simulated dataset which is used for demonstration.

Software dependency: R4.1.3
Versions the code being tested on: R4.1.3
No non-standard hardware require

Installation: Install R and Rstudio.
R could be downloaded from https://cran.r-project.org/
Rstudio could be downloaded from https://posit.co/download/rstudio-desktop/

Demo:
the demoDat.rds are data for the analysis of association between total PM2.5 and PM2.5 components and the outcomes
t1_demo.rds, t2_demo.rds, t3_demo.rds are for the analysis evaluating the association between source-specific PM2.5 and the outcomes

t1_demo.rds, t2_demo.rds, t3_demo.rds are created by merging the demoDat.rds with the source-specific pm2.5 data and separated by
3 time periods (2000-2005, 2006-2010, 2011-2016).

The mainAnlysis.R inlcudes code for the main analysis and uses demoDat.rds, t1_demo.rds, t2_demo.rds, t3_demo.rds

NMF_demo.R is the demo code for how we conducted non-negative matrix factorzation.
The we also included demo PM2.5 component data (component_demo.rds)

The expected output of these code are the effect estimates (different from the results in our study because the demo data were generated randomly)

Expected run time of the data will be around 4-5 hours
