# Supplementary material for: Long-term exposure to ambient PM2.5, particulate constituents and hospital admissions from non-respiratory infection
Source: Nat Commun. 2024 Feb 19;15:1518. doi: 10.1038/s41467-024-45776-0 (PMC10876532; doi:10.1038/s41467-024-45776-0)
Supplement: Supplementary file 5 — Reporting Summary [file 41467_2024_45776_MOESM5_ESM.pdf]

nature portfolio

Corresponding author(s): Yijing Feng

Last updated by author(s): Dec 4, 2023

Reporting Summary

Nature Portfolio wishes to improve the reproducibility of the work that we publish. This form provides structure for consistency and transparency in reporting. For further information on Nature Portfolio policies, see our Editorial Policies and the Editorial Policy Checklist.

Statistics

For all statistical analyses, confirm that the following items are present in the figure legend, table legend, main text, or Methods section.

n/a	Confirmed	
[ ]	[x]	The exact sample size (*n*) for each experimental group/condition, given as a discrete number and unit of measurement
[ ]	[x]	A statement on whether measurements were taken from distinct samples or whether the same sample was measured repeatedly
[x]	[ ]	The statistical test(s) used AND whether they are one- or two-sided<br>*Only common tests should be described solely by name; describe more complex techniques in the Methods section.*
[ ]	[x]	A description of all covariates tested
[ ]	[x]	A description of any assumptions or corrections, such as tests of normality and adjustment for multiple comparisons
[ ]	[x]	A full description of the statistical parameters including central tendency (e.g. means) or other basic estimates (e.g. regression coefficient) AND variation (e.g. standard deviation) or associated estimates of uncertainty (e.g. confidence intervals)
[x]	[ ]	For null hypothesis testing, the test statistic (e.g. *F*, *t*, *r*) with confidence intervals, effect sizes, degrees of freedom and *P* value noted<br>*Give P values as exact values whenever suitable.*
[x]	[ ]	For Bayesian analysis, information on the choice of priors and Markov chain Monte Carlo settings
[x]	[ ]	For hierarchical and complex designs, identification of the appropriate level for tests and full reporting of outcomes
[x]	[ ]	Estimates of effect sizes (e.g. Cohen's *d*, Pearson's *r*), indicating how they were calculated

Our web collection on statistics for biologists contains articles on many of the points above.

Software and code

Policy information about availability of computer code

Data collection	No software was used in data collection
Data analysis	The analytic codes are available at https://github.com/yatkan/PM2.5_nonrespiratoryInfection_NC<br>The analysis was conducted using R 4.1.3<br>The required packages included tableone (0.13.2), gWQS (3.0.4), fst (0.9.8), stringr (1.5.0), sandwich (3.0-2), lmtest(0.9-40), metafor (3.8-1), dplyr (1.0.9), stats (4.1.1), ggplot2 (3.4.0), NMF (0.24.0),

For manuscripts utilizing custom algorithms or software that are central to the research but not yet described in published literature, software must be made available to editors and reviewers. We strongly encourage code deposition in a community repository (e.g. GitHub). See the Nature Portfolio guidelines for submitting code & software for further information.

Data

Policy information about availability of data

All manuscripts must include a data availability statement. This statement should provide the following information, where applicable:

- Accession codes, unique identifiers, or web links for publicly available datasets
- A description of any restrictions on data availability
- For clinical datasets or third party data, please ensure that the statement adheres to our policy

The data that support the findings of this study are available to researchers from Center for Medicare and Medicaid Services (CMS). Researchers can submit their

data request to CMS and the request will be forwarded to the CMS dissemination contractor for processing. Processing of the data takes approximately 2-4 weeks (depending on the number and years of files being requested). However, the data use agreement prevents us from sharing that data and so are not publicly available. According to the DUA, our group could only access the data on the level 3 cluster of Harvard University with controlled access and cannot download the data.
The air pollution data used in this study are publicly available on the SEDAC website: https://sedac.ciesin.columbia.edu/data/set/aqdh-pm2-5-component-ec-nh4-no3-oc-so4-50m-1km-contiguous-us-2000-2019 and https://sedac.ciesin.columbia.edu/data/set/aqdh-pm2-5-component-trace-elements-50m-1km-contiguous-us-2000-2019 and https://sedac.ciesin.columbia.edu/data/set/aqdh-pm2-5-o3-no2-concentrations-zipcode-contiguous-us-2000-2016
The data from American Community Survey and US census are available at https://data.census.gov/
Data from BFRSS are available at https://www.cdc.gov/brfss/annual_data/annual_data.htm
Data from Dartmouth Healthcare Atlas are available at https://data.dartmouthatlas.org/
Source data are provided with this paper.

Research involving human participants, their data, or biological material

Policy information about studies with human participants or human data. See also policy information about sex, gender (identity/presentation), and sexual orientation and race, ethnicity and racism.

Reporting on sex and gender	Sex is included as confounder in this study. Sex information are collected by the Center for Medicare and Medicaid Services.
Reporting on race, ethnicity, or other socially relevant groupings	race is included as a confounder in this study. Information are collected by the Center for Medicare and Medicaid Services. Given that this is a ecological study, we calculated the proportion of white and black participants for each ZIP code and used the proportion as a confounder in the analysis.
Population characteristics	The study population is Medicare beneficiaries aged 65 or older between 2000-2016
Recruitment	The Medicare FFS beneficiaries who had record in the denominator files are automatically included in this study
Ethics oversight	The study is approved by the Harvard Institutional Review Board

Note that full information on the approval of the study protocol must also be provided in the manuscript.

Field-specific reporting

Please select the one below that is the best fit for your research. If you are not sure, read the appropriate sections before making your selection.

☐ Life sciences ☐ Behavioural & social sciences ☒ Ecological, evolutionary & environmental sciences

For a reference copy of the document with all sections, see nature.com/documents/nr-reporting-summary-flat.pdf

Ecological, evolutionary & environmental sciences study design

All studies must disclose on these points even when the disclosure is negative.

Study description	This is a cohort study evaluating the association between PM2.5, PM2.5 constituents and hospital admissions from non-respiratory infection. The exposure of the study was PM2.5 and its constituents, this is an observational study, such that there is no intervention. The study unit in here is ZIP code-year. After data aggregation, we included data from 436,577 ZIP code-years in the analysis.
Research sample	The study population are Medicare beneficiaries aged 65 or older. The data was obtained from CMS. We chose this population because hospital admission data were available from this population and that the data covers almost all the ares in the US. Moreover, the older population is a vulnerable group to air pollution and infections. This study sample is meant to represent the older populations in the US.
Sampling strategy	We included Medicare Fee For Service beneficiaries between 2000-2016 who resided in ZIP codes with more than 100 Medicare beneficiaries. We only included beneficiaries between 2000-2016 because our exposure data was available for 2000-2019 while our Medicare data was only available up to 2016. We only included those who came from ZIP codes with more than 100 beneficiaries because we were running the analysis at ZIP code level. Cases are likely to occur in ZIP code with more beneficiaries and that including ZIP codes with small number of beneficiaries could induce noise to our analysis. We did not calculate the sample size specifically for this study. However, the study includes data from 67,005,279 Medicare beneficiaries, which is a really large sample size. Such that we believe that the sample size is sufficient for our research problem.
Data collection	We obtained the data of Medicare beneficiaries by submitting data request to CMS. No direct data collection was conducted by our side.<br>For exposure data, we obtained the monitor data from the US Environmental Protection Agency. After obtaining the data, our group estimated the air pollution exposure across the whole US using ensemble machine learning models.
Timing and spatial scale	This study covers the contiguous US between Jan 1st 2000-Dec 31st 2016. We only included the data from contiguous US because our exposure data was only estimated for the contiguous US. We conducted our analysis using data between Jan 1st 2000-Dec 31st 2016 because our exposure data was available for 2000-2019 while our Medicare data was only available up to 2016. The spatial scale was at ZIP code level because the we did not have the exact address for the Medicare beneficiaries and only ZIP code information was available.

Data exclusions	We excluded data from ZIP codes with less than 100 Medicare FFS beneficiaries because we were running the analysis at ZIP code level. Cases are likely to occur in ZIP code with more beneficiaries and that including ZIP codes with small number of beneficiaries could induce noise to our analysis.
Reproducibility	No experiment was conducted
Randomization	There was no randomization because this is an observational study and there was no intervention.
Blinding	There was no binding because this is an observational study and there was no intervention to blind the participants from.

Did the study involve field work? ☐ Yes ☒ No

Reporting for specific materials, systems and methods

We require information from authors about some types of materials, experimental systems and methods used in many studies. Here, indicate whether each material, system or method listed is relevant to your study. If you are not sure if a list item applies to your research, read the appropriate section before selecting a response.

Materials & experimental systems

n/a	Involved in the study	
☒	☐	Antibodies
☒	☐	Eukaryotic cell lines
☒	☐	Palaeontology and archaeology
☒	☐	Animals and other organisms
☒	☐	Clinical data
☒	☐	Dual use research of concern
☒	☐	Plants

Methods

n/a	Involved in the study	
☒	☐	ChIP-seq
☒	☐	Flow cytometry
☒	☐	MRI-based neuroimaging

Plants

Seed stocks	*Report on the source of all seed stocks or other plant material used. If applicable, state the seed stock centre and catalogue number. If plant specimens were collected from the field, describe the collection location, date and sampling procedures.*
Novel plant genotypes	*Describe the methods by which all novel plant genotypes were produced. This includes those generated by transgenic approaches, gene editing, chemical/radiation-based mutagenesis and hybridization. For transgenic lines, describe the transformation method, the number of independent lines analyzed and the generation upon which experiments were performed. For gene-edited lines, describe the editor used, the endogenous sequence targeted for editing, the targeting guide RNA sequence (if applicable) and how the editor was applied.*
Authentication	*Describe any authentication procedures for each seed stock used or novel genotype generated. Describe any experiments used to assess the effect of a mutation and, where applicable, how potential secondary effects (e.g. second site T-DNA insertions, mosiacism, off-target gene editing) were examined.*
